# Microfluidic Process Intensification for the Synthesis of Nitrogen-Containing Bio-Based Polyols via Aminolysis of Epoxidized Soybean Oil

**DOI:** 10.3390/polym18182217

**Published:** 2026-09-11

**Authors:** Ke Yang, Xue Li, Jian Wang, Zhenhua Sun, Yanru Chen, Jiangkai Qiu, Qingli Cheng

**Affiliations:** 1 State Key Laboratory of Chemical Safety, Sinopec Research Institute of Safety Engineering Co., Ltd., Qingdao 266000, China; 2 Technical Testing Center of Shengli Oilfield Branch of China Petrochemical Corporation, Dongying 257000, China; 3 State Key Laboratory of Materials-Oriented Chemical Engineering, College of Biotechnology and Pharmaceutical Engineering, Nanjing Tech University, Nanjing 211816, China

**Keywords:** epoxidized soybean oil, nitrogen-containing polyols, ring-opening reaction, microfluidic synthesis

## Abstract

A continuous-flow microfluidic strategy was developed for the synthesis of nitrogen-containing bio-based polyols via cyclohexylamine aminolysis of epoxidized soybean oil (ESO). The effects of temperature, residence time, catalyst loading, flow rate, and channel diameter on epoxy and hydroxyl values were systematically investigated. Under optimal conditions (100 °C, 60 min, 5 wt% catalyst), a hydroxyl value of 259 mg_KOH_/g and a residual epoxy value of 0.11% were achieved. Compared to a conventional batch process requiring 12 h, the microfluidic method shortened the reaction time to minutes while delivering significantly higher hydroxyl values and narrower molecular weight distribution. FTIR, GPC, DSC, and TGA analyses confirmed more complete conversion, suppressed etherification side reactions, and superior structural uniformity in the microreactor-derived products. Based on the experimental results, microfluidic processing showed the potential for efficient and sustainable production of nitrogen-containing bio-based polyols.

## 1. Introduction

Nitrogen-containing polyols constitute a vital class of fine chemical intermediates with indispensable roles in the pharmaceutical, agrochemical, high-performance polymer, and advanced functional material sectors [1,2]. Currently, industrial synthetic routes to nitrogen-containing polyols are heavily reliant on fossil-derived feedstocks. Traditional processes based on non-renewable resources not only typically entail harsh reaction conditions, such as elevated temperatures and pressures, but also generate substantial quantities of environmentally detrimental byproducts and waste, thereby posing a significant challenge to ecological sustainability [3,4,5]. Consequently, the development of novel strategies for producing nitrogen-containing polyols from renewable biomass feedstocks is of paramount strategic importance for reducing dependence on fossil products and mitigating environmental pressures [6,7]. This endeavor has thus emerged as a frontier research topic within the domain of biomass valorization.

Among diverse biomass feedstocks, bio-based polyols from vegetable oils are regarded as some of the most promising alternatives to petroleum-based polyols, owing to their low cost, vast global abundance, inherent renewability, and the unique chemical modifiability of their molecular architecture [8,9,10]. Current synthetic approaches to bio-based polyols from vegetable oils encompass multiple technical pathways, among which the epoxide ring-opening method has garnered considerable attention due to its relatively straightforward process flow, pronounced cost-effectiveness, and favorable operational safety profile [11,12]. This methodology generally follows a two-step strategy: initial quantitative epoxidation of the carbon–carbon double bonds within the triglyceride backbone to yield epoxidized vegetable oil, followed by selective nucleophilic attack on the oxirane rings to introduce hydroxyl functionalities [13]. While the epoxidation step has been extensively investigated, the choice of nucleophilic reagent in the subsequent ring-opening step remains decisive in dictating critical product parameters such as hydroxyl value, viscosity, and average functionality [14,15]. ESO, a bulk chemical derivative of soybean oil, exhibits a suite of exceptional attributes including high thermal stability, excellent photostability, broad matrix compatibility, low-temperature flexibility, low volatility, and non-toxicity. It is widely employed not only as a plasticizer and thermal stabilizer [16,17] but also demonstrates substantial promise as a polymer building block [18,19,20,21]. Nonetheless, extant research on ESO ring-opening hydroxylation has predominantly centered on nucleophiles such as water, alcohols, or acrylic acid [22,23,24], whereas exploration of aminolysis systems utilizing amine-based nucleophiles remains comparatively limited. Notably, nitrogen-containing soybean oil-based polyols with controllable hydroxyl values were recently synthesized via the ring-opening of ESO with diethanolamine under specific acid catalysis [25]. This seminal finding provides essential theoretical substantiation and a foundational precedent for amine-mediated synthetic pathways toward nitrogen-containing bio-based polyols.

In recent years, microfluidic technology has emerged as a transformative process intensification platform, demonstrating immense utility in the realm of chemical synthesis [26]. The continuous-flow channels, characterized by dimensions on the micrometer scale, impart microreactors with extraordinarily high surface-area-to-volume ratios, thereby enabling dramatic enhancements in mass and heat transfer phenomena [27,28,29]. Furthermore, microfluidic technology affords the unique capability for precise spatiotemporal control over reaction parameters and seamlessly supports continuous operational modes [30,31]. These intrinsic advantages facilitate chemical transformations that proceed with greater rapidity, efficiency, and reproducibility, substantially mitigating challenges associated with process scale-up and offering a paradigm-shifting opportunity for the development of greener and more sustainable chemical processes [32,33].

Against the aforementioned research backdrop, a novel synthetic pathway toward nitrogen-containing bio-based polyols was explored in this work [34,35,36]. The key innovation of this study lies in the first application of a microfluidic strategy to the cyclohexylamine aminolysis of ESO, which not only accelerates the reaction but, as will be shown, critically suppresses etherification side reactions, leading to products with narrower molecular weight distribution. A continuous and precisely controlled reaction process was implemented in a microchannel reactor. Critical operational parameters, including reaction temperature, fluid flow rate, and residence time, were systematically optimized. Through such optimization, precise regulation of key product specifications was anticipated.

## 2. Materials and Methods

### 2.1. Materials and Reagents

All chemicals were used as received. ESO (Purity: 99%) with an epoxy content of 6.2% was purchased from Shanghai Dibai Biotechnology Co., Ltd. (Shanghai, China). Boron trifluoride diethyl etherate (purity: 99%), acetone (purity: 99%), sodium bicarbonate (purity: >99%), sodium chloride (purity: >99%), ethyl acetate (purity: >99%), p-xylene (purity: >99%), and cyclohexylamine (purity: 99.5%) were sourced from Macklin Biochemical Technology Co., Ltd. (Shanghai, China).

### 2.2. Characterization Methods

A Fourier-transform Infrared Spectrometer (FTIR, Nicolet 6700, Thermo Fisher Scientific, Waltham, MA, USA) fitted with a diamond ATR cell was used to obtain infrared spectra of absorption of the materials. Molecular weight (*M_n_*) and molecular weight distribution (*MWD*) were measured using Gel Permeation Chromatography (GPC, Waters 2414, Milford, MA, USA) at a flow rate of 1.0 mL/min in THF at 35 °C with polystyrene calibration. Thermal properties were characterized using a Differential Scanning Calorimeter (DSC, Q2000, TA, New Castle, DE, USA) and a Thermal Gravimetric Analyzer (TGA, Q500, TA, New Castle, DE, USA) under nitrogen with the heating rate of 10 °C/min. The determination of the epoxy value and the hydroxyl value refer to Standard GB/T 1677 [37] and Standard GB/T 12008 [38], respectively. ^1^H NMR and ^13^C NMR spectra were recorded on a Bruker Avance III 400 MHz nuclear magnetic resonance spectrometer (Bruker, Billerica, MA, USA) at room temperature. Deuterated chloroform (CDCl_3_) was used as the solvent, and tetramethylsilane (TMS) was adopted as the internal standard for chemical shift calibration.

### 2.3. Batch Synthesis of Nitrogen-Containing Bio-Based Polyols

ESO (1.0 equiv) was placed in a 100 °C oil bath for 5 min. The nitrogen-containing ring-opening agent (cyclohexylamine, 10.0 equiv) and boron trifluoride diethyl etherate as catalyst were added. The mixture was stirred at 100 °C for 12 h. After cooling, the product was extracted with saturated Na_2_CO_3_ solution, washed with distilled water to neutrality, dried over anhydrous Na_2_SO_4_, and excess ring-opening agent was removed under reduced vacuum to obtain the polyol.

### 2.4. Microfluidic Synthesis of Nitrogen-Containing Bio-Based Polyols

**Figure 1:**
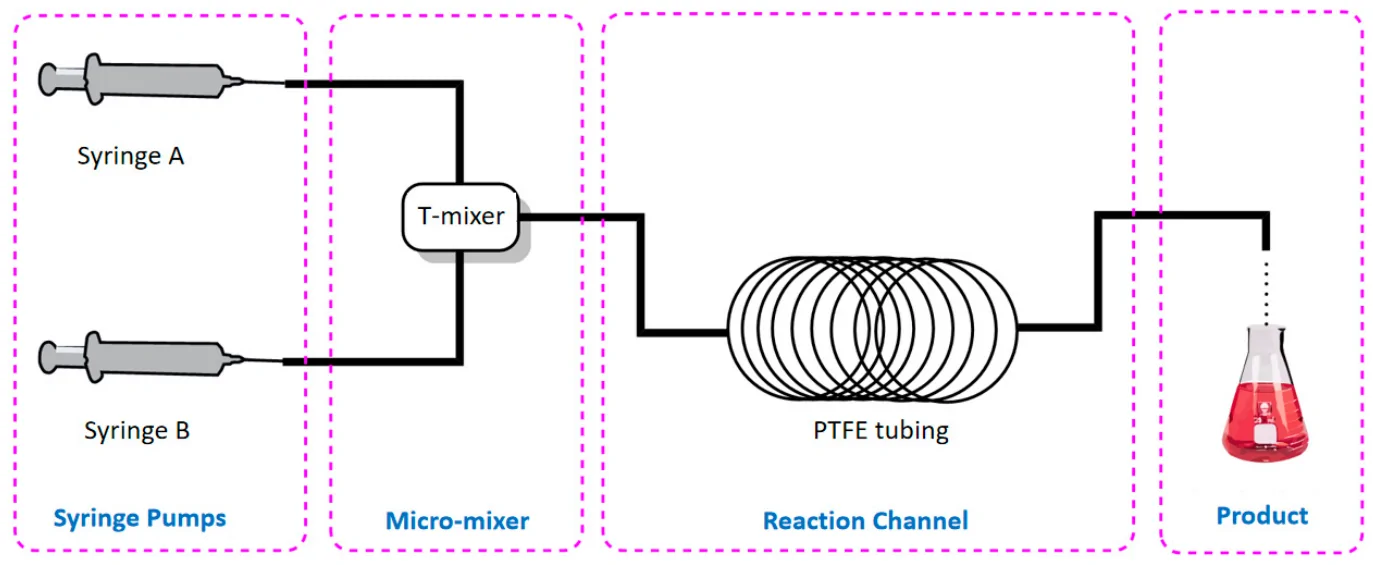
Scheme of microfluidic synthesis.

The setup consisted of syringe pumps (TYD02, Lead Fluid, Baoding, China), a T-mixer (lab-designed), PTFE tubing (inner diameters: 0.6, 1.0, 1.5 mm), and a heater (RCT5, IKA, Staufen im Breisgau, Germany). Dual-Feed Mode: Syringe A contained ESO (1.0 equiv). Syringe B contained the ring-opening agent (cyclohexylamine, 10.0 equiv) and boron trifluoride diethyl etherate as catalyst. The streams were pumped separately, metered, mixed at a junction, and reacted in the heated microchannel (Figure 1 and Figure S1, ESI). In this modes, the effluent was collected and processed similarly to the batch method.

## 3. Results and Discussion

**Figure 2:**
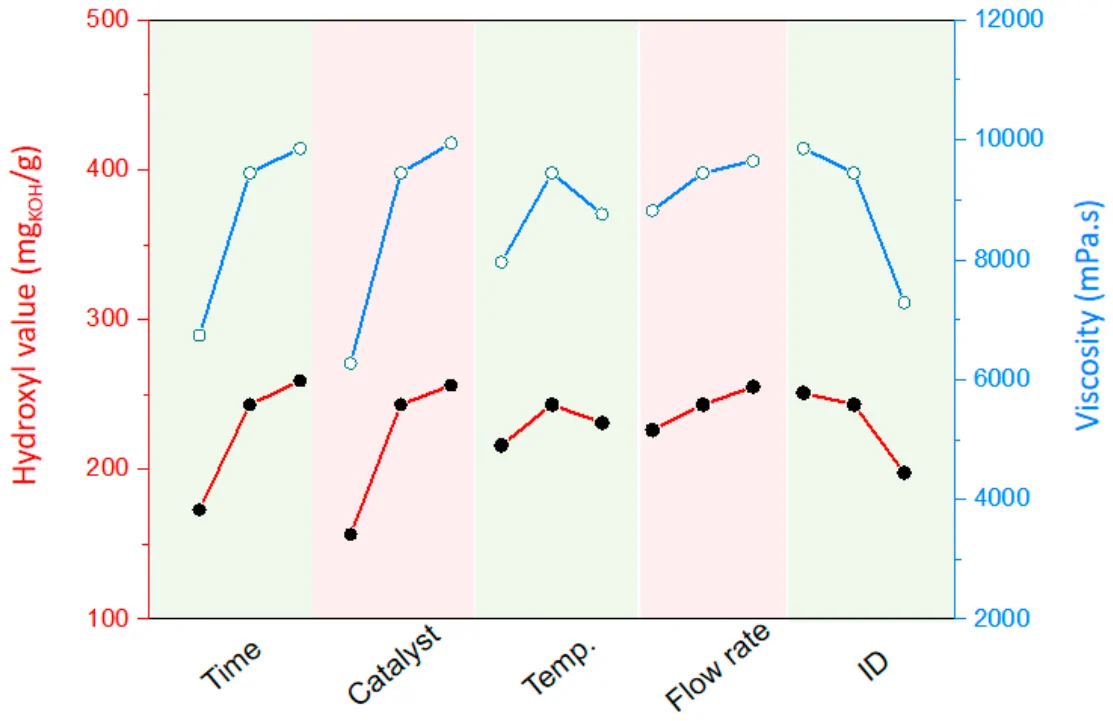
The influence of different process parameters on the hydroxyl value and viscosity of the product (red line: hydroxyl value; blue line: viscosity).

In the experiments about the preparation of bio-based polyols from ESO (Table 1), the effects of varying parameters, including inner diameter (ID) of the reaction tube, fluid flow rate, residence time, catalyst loading, and temperature, on the epoxy value and hydroxyl value were systematically investigated (Figure 2). The objective was to identify the critical conditions for process optimization.

An extension of the residence time from 20 min to 60 min resulted in a sharp decline in the epoxy value from 2.10% to 0.11% (Entries 1, 2, and 3). Concurrently, the hydroxyl value increased from 173 mg_KOH_/g to 259.3 mg_KOH_/g. These findings indicated that residence time served as a pivotal factor in determining the extent of reaction. A short residence time (20 min) led to incomplete conversion, leaving a substantial fraction of epoxy groups unopened and yielding a low hydroxyl value. With prolonged time, the reaction approached completion, and a maximum hydroxyl value was attained. At 60 min, the reaction was essentially complete, as evidenced by the residual epoxy value of merely 0.11%.

At a fixed residence time of 40 min (Entries 4, 2, and 5), the catalyst loading was increased from 1% to 5% and subsequently to 10%. The data revealed that at 1% catalyst loading, the reaction proceeded inadequately (epoxy value of 2.6%, hydroxyl value of 156.6 mg_KOH_/g). An increase in loading to 5% led to a substantial enhancement in reaction efficiency (hydroxyl value of 243 mg_KOH_/g). However, a further increase to 10% produced only a marginal improvement in the hydroxyl value (256 mg_KOH_/g) relative to the 5% condition, accompanied by an exceedingly low residual epoxy value (0.18%). This observation suggested the existence of an optimal range for catalyst loading; a loading of 5% was deemed more appropriate for the system under investigation.

When the temperature was raised from 80 °C to 100 °C (Entries 2, 7), the epoxy value remained in a comparably low range (0.42~0.52%), while the hydroxyl value rose correspondingly from 216.2 mg_KOH_/g to 243.2 mg_KOH_/g. Upon further heating to 120 °C (Entry 6), the epoxy value dropped sharply to 0.06%, indicating nearly complete consumption of the epoxy groups. However, the hydroxyl value did not continue to increase but instead decreased to 231.1 mg_KOH_/g, and viscosity remained unchanged or even increased slightly. This trend conformed to the Arrhenius equation, whereby elevated temperatures significantly accelerate the reaction kinetics and drive the reaction toward higher conversion. But excessively high temperatures may induce side reactions such as dehydration of polyols, thereby expending some of the hydroxyl groups already formed.

For the 1 mm inner diameter tubing, the flow rate was varied from 0.058 mL/min to 0.234 mL/min (Entries 9, 2, and 8) while the tube length was proportionally adjusted to maintain a constant residence time of 40 min. The hydroxyl value increased progressively from 226.4 mg_KOH/_g at the lowest flow rate to 243.2 mg_KOH_/g at the intermediate rate, and further to 255.2 mg_KOH_/g at the highest flow rate. Correspondingly, the residual epoxy value declined from 0.90% to 0.52% and then to 0.21%. Since residence time was held constant, the observed enhancement in reaction extent could be attributed to improved mixing efficiency at elevated flow rates. Higher volumetric flow rates increased the linear velocity within the microchannel, thereby intensifying radial convective mass transfer and reducing concentration gradients across the channel cross-section. This facilitated more efficient contact between the reactants and catalyst, leading to a more complete ring-opening reaction. In contrast, at lower flow rates, the prevailing laminar flow conditions may have permitted the development of broader concentration boundary layers, thereby limiting the overall reaction rate despite an identical residence time. These results underscored the critical role of flow-induced mixing in determining microfluidic reaction performance when residence time was decoupled from the flow rate through proportional adjustment of the channel length.

Under a constant volumetric flow rate (0.117 mL/min), the tubing inner diameter was varied from 0.6 mm to 1.5 mm, and the tubing length was proportionally adjusted to maintain a constant residence time. The resulting hydroxyl value for the 1 mm diameter tubing (243 mg_KOH_/g, Entry 2) was found to be higher than that for the 1.5 mm diameter tubing (197.7 mg_KOH_/g, Entry 11). Although the hydroxyl value for the 0.6 mm diameter tubing (251.1 mg_KOH_/g, Entry 10) exceeded that of Entry 2, its lower epoxy value indicated a more advanced reaction. Tubing diameter directly affected the specific surface area and shear rate within the microchannel. Smaller diameters (0.6 mm) offered larger specific surface areas and superior mass transfer, which could potentially favor the reaction. However, excessively small diameters may lead to significantly increased pressure drop and susceptibility to clogging. Larger diameters (1.5 mm) diminished the mixing intensification advantages inherent to microfluidic fields, thereby reducing reaction efficiency. This interpretation was consistent with the elevated residual epoxy value (1.6%) and the lowest hydroxyl value (197.7 mg_KOH_/g) observed in Entry 11. Under the present experimental conditions, the 1 mm diameter tubing appeared to provide a favorable balance between mass transfer efficiency and operational feasibility.

**Figure 3:**
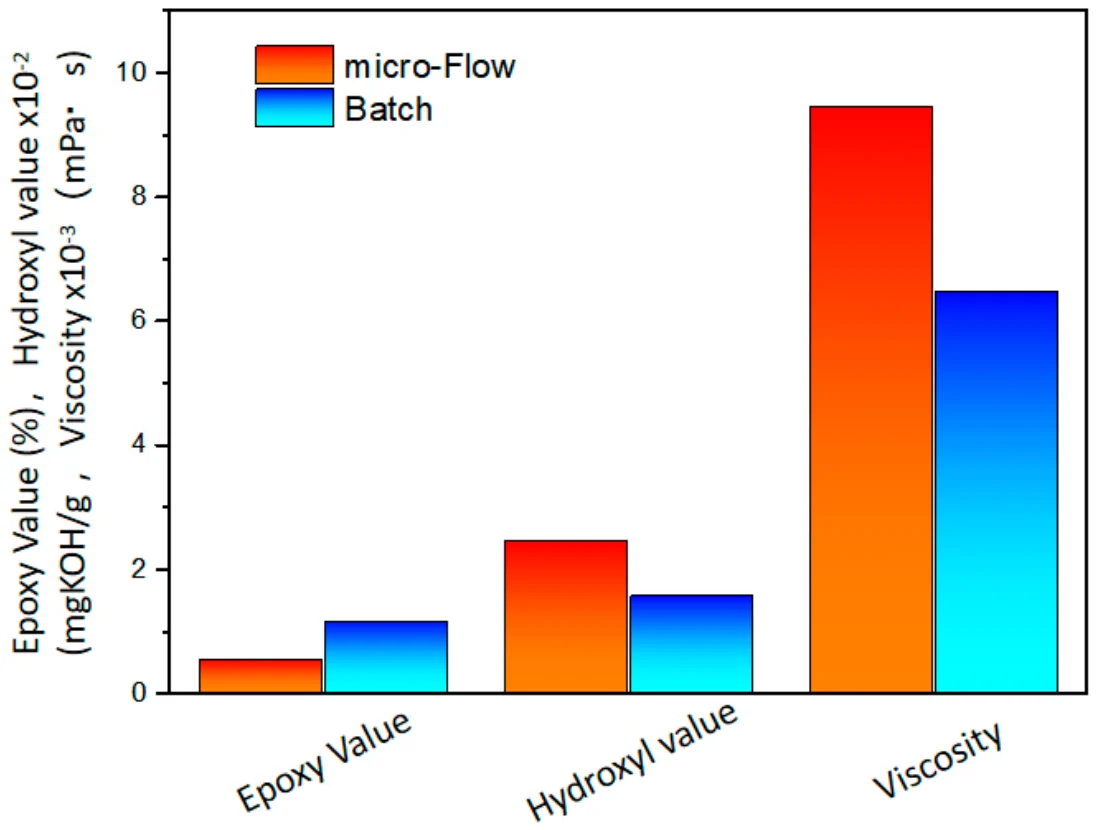
The epoxy value, hydroxyl value, and viscosity of obtained product in different reactors.

The most direct comparison (Figure 3) was drawn between Entry 12 (batch process) and Entry 2 (microfluidic process under baseline conditions). Under identical catalyst loading (5 wt%) and reaction temperature (100 °C), the batch process required a reaction time of 720 min (12 h). The resulting product exhibited a residual epoxy value of 1.15% and a hydroxyl value of 155.1 mg_KOH_/g. In stark contrast, the microfluidic process, with a residence time of merely 40 min, yielded a product with a substantially reduced epoxy value (0.5%) and a markedly enhanced hydroxyl value (243 mg_KOH_/g). Under batch conditions, increasing the stirring rate failed to further improve the ring-opening efficiency (Entry 14), and shortening the reaction time likewise led to a further decrease in ring-opening efficiency (Entry 13). This comparison provided unequivocal evidence that microfluidic technology, by virtue of its exceptionally high specific surface area, dramatically intensified mass and heat transfer efficiency. The rate of epoxy ring-opening was lowered by a factor of 10~20, effectively reducing the required reaction time from the hour scale to the minute scale. Simultaneously, a product with a higher degree of conversion (lower residual oxirane content) and greater functionality (higher hydroxyl value) was obtained. These findings establish a robust foundation for the efficient, continuous production of bio-based polyols. Notably, the microreactor setup can be operated continuously for 12 h to yield 45 g of the corresponding product.

**Figure 4:**
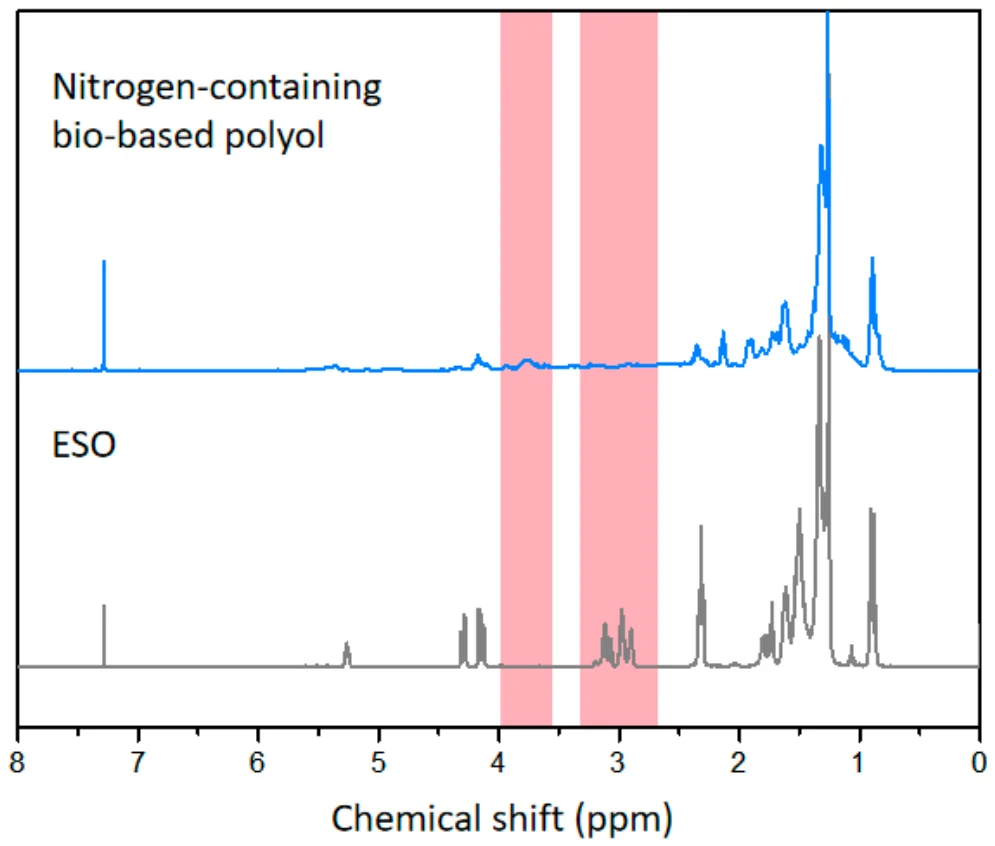
^1^H NMR curves of obtained product in different reactors.

The ^1^H NMR spectra (Figure 4) clearly demonstrated a significant reduction in the epoxy proton signals (2.8~3.1 ppm) relative to the starting epoxidized soybean oil, confirming successful ring opening, and the appearance of characteristic cyclohexyl proton peaks (1.8 ppm) and α-methine protons adjacent to nitrogen and oxygen (3.8 ppm), consistent with the formation of β-aminoalcohol structures. ^13^C NMR spectra could also prove similar conclusions (Figure S2, ESI).

**Figure 5:**
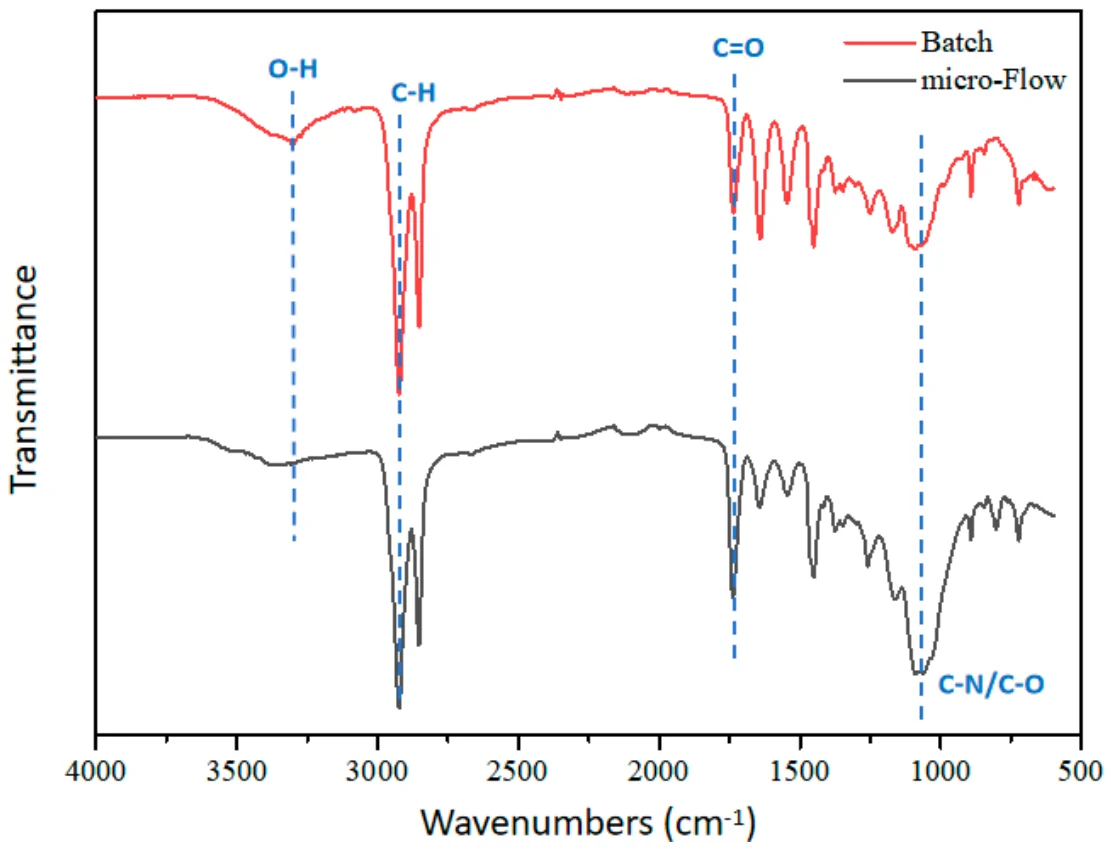
FTIR curves of obtained products in different reactors.

The FTIR spectra of the obtained products synthesized in the microfluidic reactor and the batch reactor exhibited a high degree of consistency in the principal functional group regions (Figure 5). Main absorption peaks were observed at approximately 3300 cm^−1^ (O-H stretching vibration of hydroxyl groups), 2900 cm^−1^ (C-H stretching vibration of alkyl groups), and 1700 cm^−1^ (C=O stretching vibration of ester carbonyl groups). These observations confirmed that the amine-induced ring-opening reaction of ESO proceeded successfully in both reactor configurations. Nevertheless, discernible differences were noted in the fingerprint region spanning 1500–500 cm^−1^. The spectral bands in the 1050–1150 cm^−1^ interval, attributed to C-O and potentially C-N stretching vibrations, were more pronounced for the microreactor-derived product. This suggested a higher content of targeted C-O/C-N bonds or a greater extent of ring-opening conversion in the microreactor product.

The analysis of the GPC elution profiles revealed distinct differences in the *M_n_* characteristics of the products obtained from the two reactor configurations (Figure S3, ESI). The polyol synthesized via the conventional batch reactor exhibited *M_n_* of 1380 g/mol and *MWD* of 1.39. In contrast, the product derived from the microfluidic reactor displayed a lower *M_n_* of 1100 g/mol and significantly narrower *MWD* of 1.18. The reduced *M_n_* and the constrained *MWD* observed for the microfluidic product indicated a lower extent of undesired side reactions during the ring-opening process.

**Figure 6:**
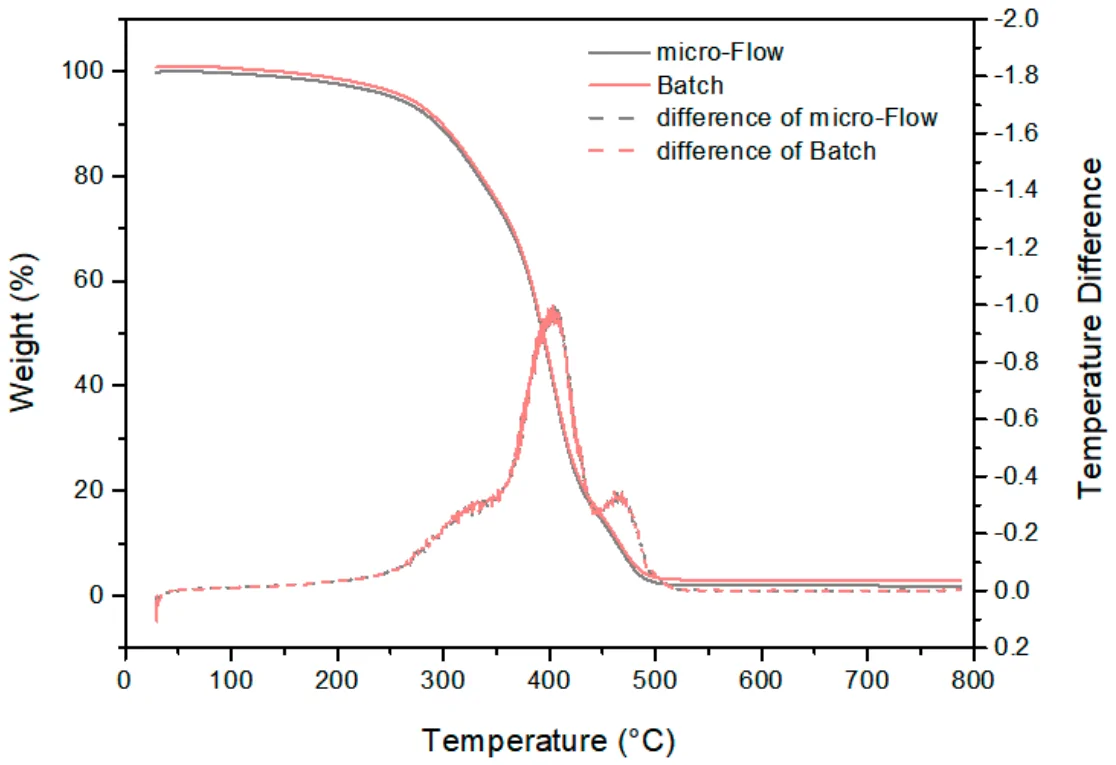
TGA curves of obtained products in different reactors.

The DSC analysis (Figure S4, ESI) demonstrated that the heat flow curve of the microreactor-derived product exhibited more consolidated thermal behavior (Tg_flow_ = 58.4 °C vs. Tg_batch_ = 59.0 °C). A more well-defined onset temperature for phase transition and diminished signal fluctuation were observed. In comparison, the DSC curve of the batch reactor product appeared comparatively diffuse. This contrast directly reflected the core advantage of the microreactor in intensifying mass transfer and maintaining homogeneity within the reaction environment. Consequently, a product with a more consistent molecular structure and compositional distribution was generated. Batch reactions, constrained by limited macroscopic mixing efficiency and potential local fluctuations in temperature or concentration, yielded products with greater microstructural dispersity. This dispersity manifested as larger variability in the observed thermal behavior. The TGA curves indicated that the thermal decomposition behaviors of the products from both reactor types were broadly similar (Figure 6). The primary decomposition process occurred in three distinct stages, with characteristic decomposition temperatures located near 320 °C, 403 °C, and 470 °C, respectively. This finding suggested that although differences in microstructural uniformity existed (as evidenced by DSC), the fundamental chemical composition and the overarching thermal stability framework of the polyol backbone remained unaltered by the choice of reactor type.

The advantages of microfluidic technology over conventional batch processing were clearly demonstrated through comprehensive product characterization. FTIR spectroscopy confirmed successful amine-induced ring-opening in both systems, yet the microreactor-derived product exhibited enhanced spectral features in the C-O/C-N stretching region, indicating more complete conversion to the targeted hydroxyl and amine functionalities. The GPC analysis revealed a lower *M_n_* and a markedly narrower *MWD* for the microfluidic product, signifying effective suppression of undesired etherification oligomerization. DSC thermograms further highlighted a more uniform thermal response with sharper phase-transition onset, reflecting superior microstructural homogeneity achieved under the precisely controlled laminar flow conditions of the microchannel. From spectroscopic, chromatographic, and thermal analyses, the collective evidence affirmed that the intensified mass and heat transfer, precise spatiotemporal regulation, and minimized back-mixing inherent to microfluidic processing collectively enabled a more selective, efficient, and reproducible epoxy ring-opening reaction.

## 4. Conclusions

Through systematic investigation of microfluidic process parameters, it was concluded that microfluidic reaction technology offered an enhancement in efficiency for the preparation of bio-based polyols from ESO, relative to conventional batch processing. The core advantage resided in the microscale confinement effect, which enabled instantaneous uniform mixing of reactants and precise temperature control. Consequently, the required reaction time was reduced by an order of magnitude. For the targeted hydroxyl value, reaction temperature, residence time, and catalyst loading were identified as the critical determinants. In the present system, a combination of 100 °C and 60 min approached complete conversion. An economically optimal catalyst loading (5%) was established. The ESO-cyclohexylamine system was found to be profoundly influenced by hydrodynamic parameters such as tubing diameter and flow rate. Their coordinated optimization was therefore required. Notably, even under non-optimal parameter combinations (e.g., Entries 3, 4, and 11), the hydroxyl values obtained via microfluidic processing within extremely short durations (20–40 min) approached or exceeded those achieved after 12 h of batch reaction (155.1 mg _KOH_/g). This observation strongly corroborated the immense potential of microfluidic technology for intensifying this specific ring-opening reaction.

## Figures and Tables

**Table 1:** Synthesis of nitrogen-containing bio-based polyols in the continuous flow and batch reaction conditions.

Entry	ID (mm)	Flow Velocity (mL/min)	Time (min)	Catalyst (wt%)	Temp. (°C)	Epoxy Value (%)	Hydroxyl Value (mg_KOH_/g)	Average Hydroxyl Functionality ^c^ (*f*)	Viscosity (mPa‧s)
1	1.0	0.117	60	5	100	0.11	259.3	5.1	9860
2	1.0	0.117	40	5	100	0.52	243.2	4.7	9450
3	1.0	0.117	20	5	100	2.10	173.1	3.4	6740
4	1.0	0.117	40	1	100	2.60	156.6	3.1	6270
5	1.0	0.117	40	10	100	0.18	256.2	5.3	9950
6	1.0	0.117	40	5	120	0.06	231.1	4.9	9760
7	1.0	0.117	40	5	80	0.42	216.2	4.2	7960
8	1.0	0.234	40	5	100	0.21	255.2	5.2	9650
9	1.0	0.058	40	5	100	0.90	226.4	4.4	8820
10	0.6	0.117	40	5	100	0.31	251.1	4.9	9860
11	1.5	0.117	40	5	100	1.60	197.7	3.9	7290
12	Batch ^a^	-	720	5	100	1.15	155.1	3.8	6460
13	Batch ^a^	-	540	5	100	2.28	118.9	2.8	5190
14	Batch ^b^	-	720	5	100	1.20	164.5	3.8	7430

^a^ The stirring speed was 1000 rpm, ^b^ the stirring speed was 1500 rpm, ^c^ hydroxyl value × *M_n_*/56,100.

## Data Availability

The original contributions presented in the study are included in the article/Supplementary Materials. Further inquiries can be directed to the corresponding author.

## Supplementary Materials

The following supporting information can be downloaded at https://www.mdpi.com/article/10.3390/polym18182217/s1: Figure S1. The setup of microfluidic synthesis; Figure S2. ^13^C NMR curves of obtained product; Figure S3. GPC curves of obtained products in different reactors; Figure S4. DSC curves of obtained products in different reactors.
