# Supplementary material for: Microfluidic Process Intensification for the Synthesis of Nitrogen-Containing Bio-Based Polyols via Aminolysis of Epoxidized Soybean Oil
Source: Polymers (Basel). 2026 Sep 11;18(18):2217. doi: 10.3390/polym18182217 (PMC13611740; doi:10.3390/polym18182217)
Supplement: Supplementary file 1 [file polymers-18-02217-s001.zip › polymers-4427970-supplementary.pdf]

Microfluidic Process Intensification for the Synthesis of Nitrogen-Containing Bio-Based Polyols via Aminolysis of Epoxidized Soybean Oil

Ke Yang[1,*], Xue Li[2], Jian Wang[3], Zhenhua Sun[2], Yanru Chen[1], Jiangkai Qiu[3], and Qingli Cheng[1]

[1] State Key Laboratory of Chemical Safety, Sinopec Research Institute of Safety and Engineering Co., Ltd, Qingdao, 266000, China

[2] Technical Testing Center of Shengli Oilfield Branch of China Petrochemical Corporation, Dongying, 257000, China

[3] *State Key Laboratory of Materials-Oriented Chemical Engineering, College of Biotechnology and Pharmaceutical Engineering, Nanjing Tech University, Nanjing 211816, China*

* Correspondence: *kikiwow@163.com*

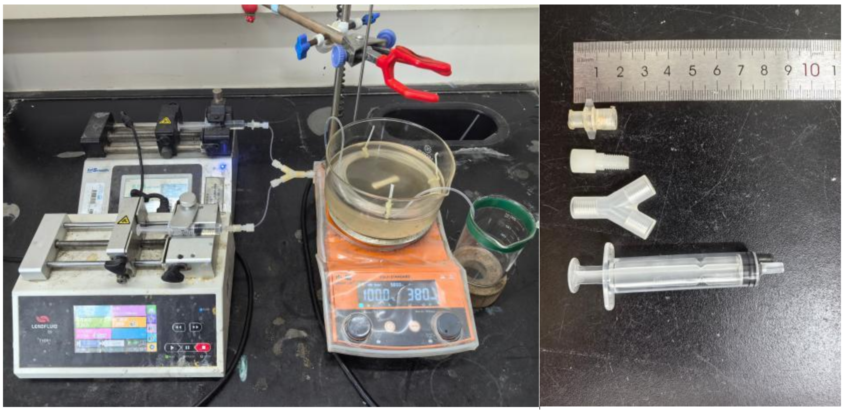

Figure S1 The setup of microfluidic synthesis

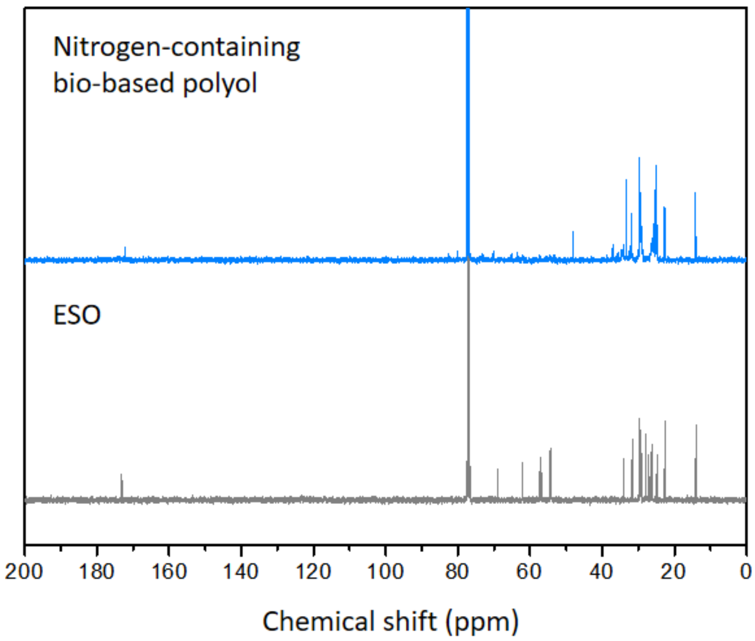

Figure S2 ^{13}C NMR curves of obtained product

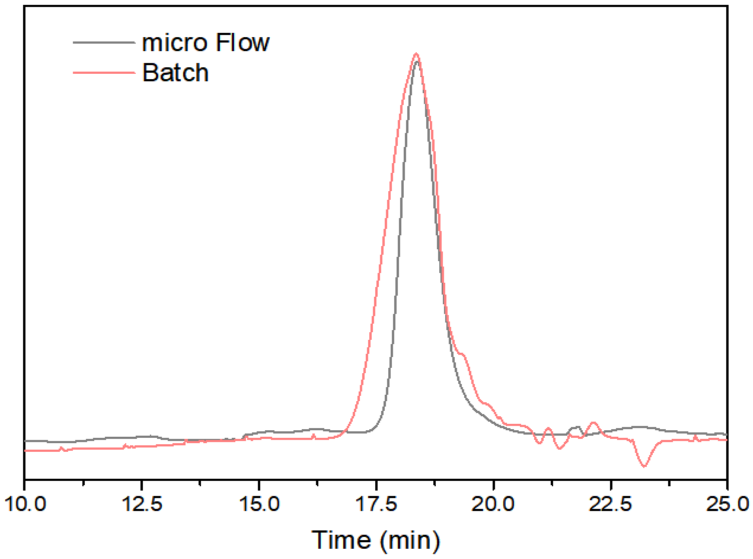

Figure S3 GPC curves of obtained products in different reactors

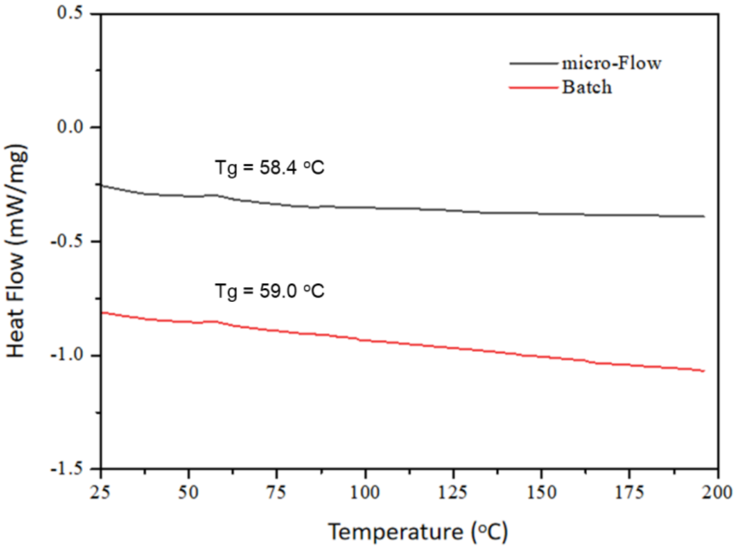

Figure S4 DSC curves of obtained products in different reactors
